# Three-dimensional (3D) brain microphysiological system for organophosphates and neurochemical agent toxicity screening

**DOI:** 10.1371/journal.pone.0224657

**Published:** 2019-11-08

**Authors:** Lumei Liu, Youngmi Koo, Chukwuma Akwitti, Teal Russell, Elaine Gay, Daniel T. Laskowitz, Yeoheung Yun

**Affiliations:** 1 FIT BEST Laboratory, Department of Chemical, Biological, and Bio Engineering, North Carolina Agricultural and Technical State University, Greensboro, North Carolina, United States of America; 2 Center for Drug Discovery, RTI International, Research Triangle Park, Durham, North Carolina, United States of America; 3 Departments of Neurology, Anesthesiology, and Neurobiology, Brain Injury Translational Research Center, Duke University, Durham, North Carolina, United States of America; Universidad de Castilla-La Mancha, SPAIN

## Abstract

We investigated a potential use of a 3D tetraculture brain microphysiological system (BMPS) for neurotoxic chemical agent screening. This platform consists of neuronal tissue with extracellular matrix (ECM)-embedded neuroblastoma cells, microglia, and astrocytes, and vascular tissue with dynamic flow and membrane-free culture of the endothelial layer. We tested the broader applicability of this model, focusing on organophosphates (OPs) Malathion (MT), Parathion (PT), and Chlorpyrifos (CPF), and chemicals that interact with GABA and/or opioid receptor systems, including Muscimol (MUS), Dextromethorphan (DXM), and Ethanol (EtOH). We validated the BMPS platform by measuring the neurotoxic effects on barrier integrity, acetylcholinesterase (AChE) inhibition, viability, and residual OP concentration. The results show that OPs penetrated the model blood brain barrier (BBB) and inhibited AChE activity. DXM, MUS, and EtOH also penetrated the BBB and induced moderate toxicity. The results correlate well with available *in vivo* data. In addition, simulation results from an *in silico* physiologically-based pharmacokinetic/pharmacodynamic (PBPK/PD) model that we generated show good agreement with *in vivo* and *in vitro* data. In conclusion, this paper demonstrates the potential utility of a membrane-free tetraculture BMPS that can recapitulate brain complexity as a cost-effective alternative to animal models.

## Introduction

Chemical agents like organophosphates (OPs) pose a grave threat to soldiers, creating an urgent need to develop better *in vitro* models for studying neurotoxicity screening, as well as developing protective measures against chemical warfare agents and therapeutic interventions. Further, more than 60,000 tons of OP pesticides are produced and used every year in the United States, which poses a significant potential health risk [1]. OPs are usually ester (R-CO_2_-R), amide (R-NH_2_), or thiol (R-SH) derivatives of phosphonic acid (H_3_O_3_P). OPs inhibit acetylcholinesterase (AChE), leading to an accumulation of acetylcholine (ACh) that induces hypercholinergic activity as a result of continuous firing between pre- and post-synaptic neurons. This process causes lethal excitotoxicity, seizures, and brain damage [2, 3]. Another set of warfare agents are chemicals that interact with GABA and/or opioid receptors, which can cause acute neurotoxicity. There is a clear need to develop a realistic *in vitro* brain microphysiological system (BMPS) that recapitulates the activities, mechanical environment, and complex physiological responses of the brain and that can be used to rapidly screen large numbers of chemical warfare compounds.

Current 2D *in vitro* models do not reliably model nor predict brain physiology and do not recapitulate the continuous interaction of multiple tissues in the central nerve system (CNS). Transwell models, which use static cultures in multiple compartments separated by a rigid, porous membrane, have limited imaging capability and vascular function. Similarly, brain slices may provide a certain level of brain circuit function, but cannot replicate the complex, multifunctional, and integrated neuro-gliovascular-immune system needed for toxicity screening. Recently, organoid and spheroid models have been used to study brain development and dysfunction. There is a clear need to develop a dynamic 3D BMPS that can provide clinically relevant information for toxicity screening. Brain tissue models must **(i)** include different cell types, such as neurons, microglia, astrocytes, and endothelial cells with host extracellular matrix (ECM), and **(ii)** be able to replicate the spatiotemporal context of original brain tissue.

In a previous study, we successfully screened four OPs for neurotoxicity using a 3D tetraculture **microphysiological** platform [4]. In this paper, we use the same platform to screen the toxicity of an expanded set of OPs and chemical agents that interact with GABA and/or opioid receptors: agonists of GABA receptors Muscimol (MUS) and Ethanol (EtOH), and glutamate (NMDA) antagonist Dextromethorphan (DXM). We evaluated the neurotoxic agents for concentration-dependent effects on: 1) overall cell viability/toxicity, 2) penetration of the agents across the model blood brain barrier (BBB), and 3) inhibition of AChE activity in target neuron cells following exposure. We correlated the *in vitro* results with available *in vivo* data for the potential use of this platform in a high-content/throughput manner. Furthermore, we developed an *in silico* physiologically-based pharmacokinetic/pharmacodynamics (PBPK/PD) model to predict effective concentrations and correlated our results with the *in vitro* and *in vivo* data.

## Material and methods

### Chemical agent preparation

OPs Chlorpyrifos (CPF), Malathion (MT), and Parathion (PT) were purchased from Sigma-Aldrich (St. Louis, MO, USA) in neat form. They were dissolved in Dimethyl sulfoxide (DMSO) first, and then diluted to final concentration of 5% DMSO in Dulbecco’s modified eagle’s medium (DMEM, ATCC, Manassas, VA, USA) and then further diluted in DMEM to concentrations of 10^−1^, 10^−3^, 10^−5^, and 10^−7^ M. Muscimol (MUS, Sigma-Aldrich, St. Louis, MO, USA) and Dextromethorphan (DXM, USP™, Rockville, MD, USA) were diluted in DMEM to concentrations of 10^−1^, 10^−2^, 10^−3^, and 10^−4^ mM. Pure Ethanol (EtOH) purchased from Sigma-Aldrich (St. Louis, MO, USA) was diluted in DMEM to concentrations of 500, 100, 20, and 5 mM. The DMSO-contained medium were used as control (CON): 5% DMSO in DMEM for OPs, DMEM for MUS, DXM and EtOH.

### Cell culture

The complete culture medium for all cell lines was DMEM supplemented with 10% fetal bovine serum (ATLANTA Biologicals, Flowery Branch, GA, USA) and 1% Penicillin/Streptomycin solution (HyClone Laboratories Inc., South Logan, UT, USA). Murine brain cell lines bEnd.3 (endothelial cells), N2a (neuroblastoma cells), and C8-D1A (astrocytes) were purchased from ATCC. Murine brain cell line BV-2 (microglia) was obtained from Dr. G. Jean Harry (National Institute of Environmental Health Sciences, Research Triangle Park, NC). All cell lines were maintained according to provided protocols and previous studies [5]. Briefly, the cells were cultured in 75 cm^2^ flasks (Corning, NY, USA) separately, and medium was refreshed every other day until the cells reached ≥ 90% confluency.

### 3D brain microphysiological system (BMPS)

The OrganoPlate (MIMETAS, Netherlands) was used to construct the 3D membrane-free microfluidic BMPS and the tissue construct method was described previously [4]. Briefly, collagen solution (Corning, Type I Rat Tail) was diluted in DMEM and the pH was neutralized to 7.0–7.4. Collagen preparation was performed on ice. N2a, C8-D1A, and BV-2 cells were re-suspended in the ECM at the following concentrations: N2a – 3.12 x 10^6^ cells/mL, C8-D1A – 3.12 x 10^6^ cells/mL, and BV-2–1.56 x 10^6^ cells/mL. The cell-ECM mixture was added to the gel lane and incubated (37°C, 5% CO_2_) for 1 h for gel polymerization. After polymerization, bEnd.3 cells were dispensed into the medium lane at a concentration of 1 × 10^7^ cells/mL in DMEM. The plate was incubated against the side of the incubator at a 75° angle for 4 h to allow bEnd.3 cells to settle against the ECM. Then, 50 μL medium was added to the medium inlet and outlet. The plate was placed on an interval rocker (MIMETAS, The Netherlands) for medium perfusion inside the incubator (37°C, 5% CO_2_). The rocker (switching between +7° and −7° inclination every 8 min) created a bi-directional flow with a mean flow rate of 2.02 μL/min and a mean shear rate of 0.13 Pa. Medium (50 μL each in the inlet and outlet) was refreshed every other day.

### Compound exposure and residual measurement

After five days of 3D co-culture, different concentrations of prepared OPs (CPF, MT, and PT), MUS, DXM, and EtOH were added to the medium inlet of the neurovascular endothelial lane (blood lane). After 24 hours, the solution was removed from the blood lane and stored at −20°C. The residual concentrations of the OPs were measured by liquid chromatography with tandem mass spectrometry (LC-MS/MS) using a triple quadrupole mass spectrometer coupled with a Shimadzu Prominence High Performance LC (HPLC) system (API 3200, AB Sciex).

### Cell viability

The LIVE/DEAD^®^ Viability/Cytotoxicity Kit for mammalian cells (Invitrogen, Carlsbad, CA) was used to test viability. After washing cells twice with PBS, cells were incubated for 30 min in 2 μM Calcein AM, 4 μM Ethidium homodimer-1 (EthD-1), and 2 μg/mL Hoechst to stain live cells green, dead cells red, and nuclei blue, respectively. Viability was calculated using ImageJ software (US National Institutes of Health, Bethesda, MD) by dividing live cell number by total cell number and multiplying by 100%.

### Acetylcholinesterase (AChE) activity assay

The Molecular Probes™ Amplex™ Acetylcholine (ACh)/Acetylcholinesterase Assay Kit was used to measure AChE activity. Briefly, standards and working solutions were prepared according to the manufacturer’s instruction. Samples and standards were incubated in working solution for 30 min. The fluorescence was read using a CLARIOstar microplate reader (BMG LABTECH, Cary, NC, USA) at 25°C.

### Data analysis

Cell viability, AChE activity, and residual concentration data (Mean ± Standard deviation) were plotted using Microsoft Excel. The correlation of *in vitro* AChE activity (inhibitor concentration 50%, IC_50_) and viability (lethal concentration 50%, LC_50_) data with *in vivo* lethal dose 50% (LD_50_) data was analyzed using Origin^®^ 2018 (OriginLab, Northampton, MA, USA).

### Physiologically-based pharmacokinetic /pharmacodynamic (PBPK/PD) model

A PBPK/PD model for AChE inhibition was developed for CPF and diisopropylfluorophosphate (DFP) using MATLAB Simbiology Toolbox. The model has multiple compartments of lung, brain, kidney, liver, diaphragm, slowly perused and rapid perfused tissues, arterial blood and venous blood. In terms of murine-based PBPK/PD modeling of DFP toxicity, this paper followed the previous work from Gearhart et. al. [6], which includes DFP tissue/blood partition coefficients, rates of DFP hydrolysis by esterases, and DFP-esterase bimolecular inhibition rate constants. The model includes two DFP-based esterase reactions: 1) A esterase (AEST) hydrolysis to the non-inhibitory product diisopropylphosphoric acid (DIP) and 2) B esterase (BEST) hydrolysis, which inhibits AChE enzymatic activity. The same determinants were used for the case of CPF and follow previous work [7]. This murine-based CPF toxicity PBPK/PD model with esterase dynamics integrates target tissue dosimetry and dynamic response (i.e. esterase inhibition) describing the uptake, metabolism, and disposition of CPF, CPF-oxon, and 3,5,6-trichloro-2-pyridinol (TCP), and the associated cholinesterase (ChE) inhibition kinetics in blood and tissue following CPF exposure. AEST is allocated to blood, liver, kidney, rapid perfusion, and brain compartments. For both OPs, the hydrolysis reaction is modeled by the Michaelis-Menten kinetics with V_max_ and K_m_ parameters measured *in-vitro*.

## Results

### Microfluidic 3D BMPS platform

Microfluidic brain tissue chips were constructed using the 2-lane OrganoPlate (MIMETAS, Netherlands), with the construct method previously described [7]. Brain tissue chips consisted of a vascular lane and brain lane with cell-gel matrix, which were separated by a capillary pressure barrier called a phase guide (Pg). The Pg separates the gel and fluid phases for membrane-free endothelial cell adhesion and BBB formation against the cell-gel matrix [8, 9]. The brain tissue constructs were placed on a rocker inside an incubator, creating dynamic medium perfusion in the vascular lane, which consisted of endothelial cells, while the brain lane consisted of neurons, astrocytes, and microglia in ECM.

### OP toxicity with different exposure times

**Fig 1:**
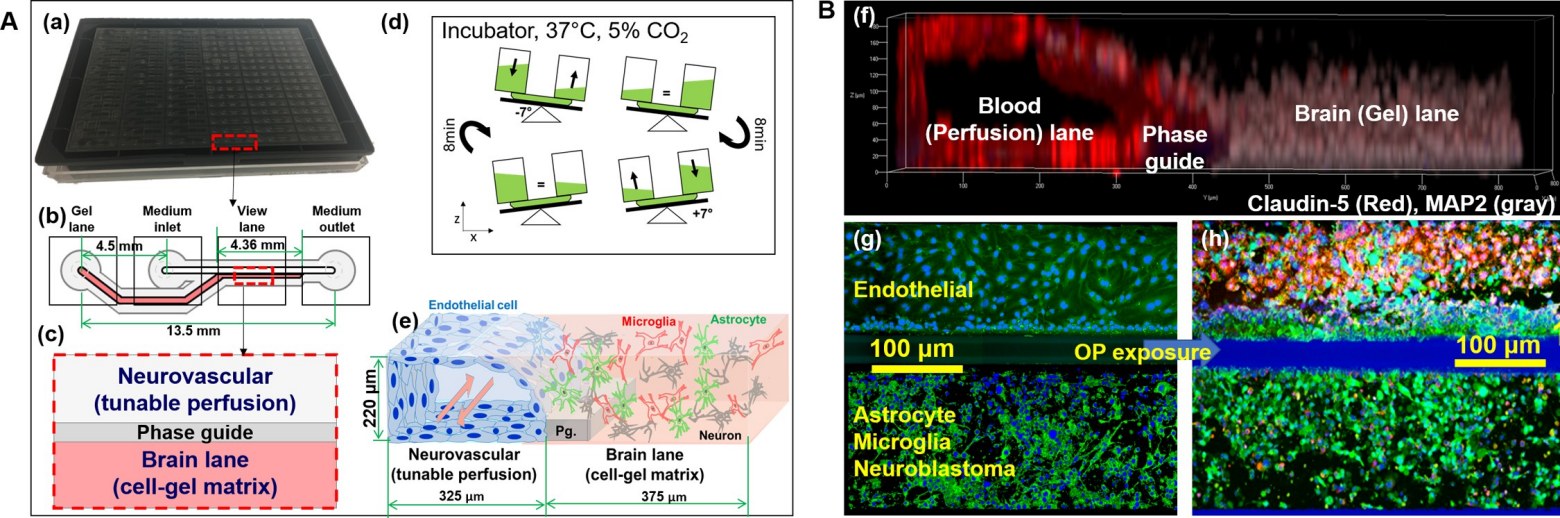
Brain microphysiological system (BMPS). (A) BMPS platform design. (a) A Mimetas OrganoPlate consisting 384 wells; (b) The schematic structure of two-lane microfluidic chamber consisting 4 wells; (c) The schematic design (top view) of the observation column; (d) The incubation of OrganoPlate in a dynamic perfusion condition; (e) Expecting cells co-culture after incubation with 220 μm height, 325 μm perfusion lane and 375 μm brain lane. (B) Immunofluorescent images of cells co-culture. (f) Side view of one microfluidic chamber. (g) Live (green)/dead (red) images before OP exposure (nucleus are stained by Hoechst to blue) and (h) after OP exposure (nucleus are stained by Hoechst to blue).

**Fig 2:**
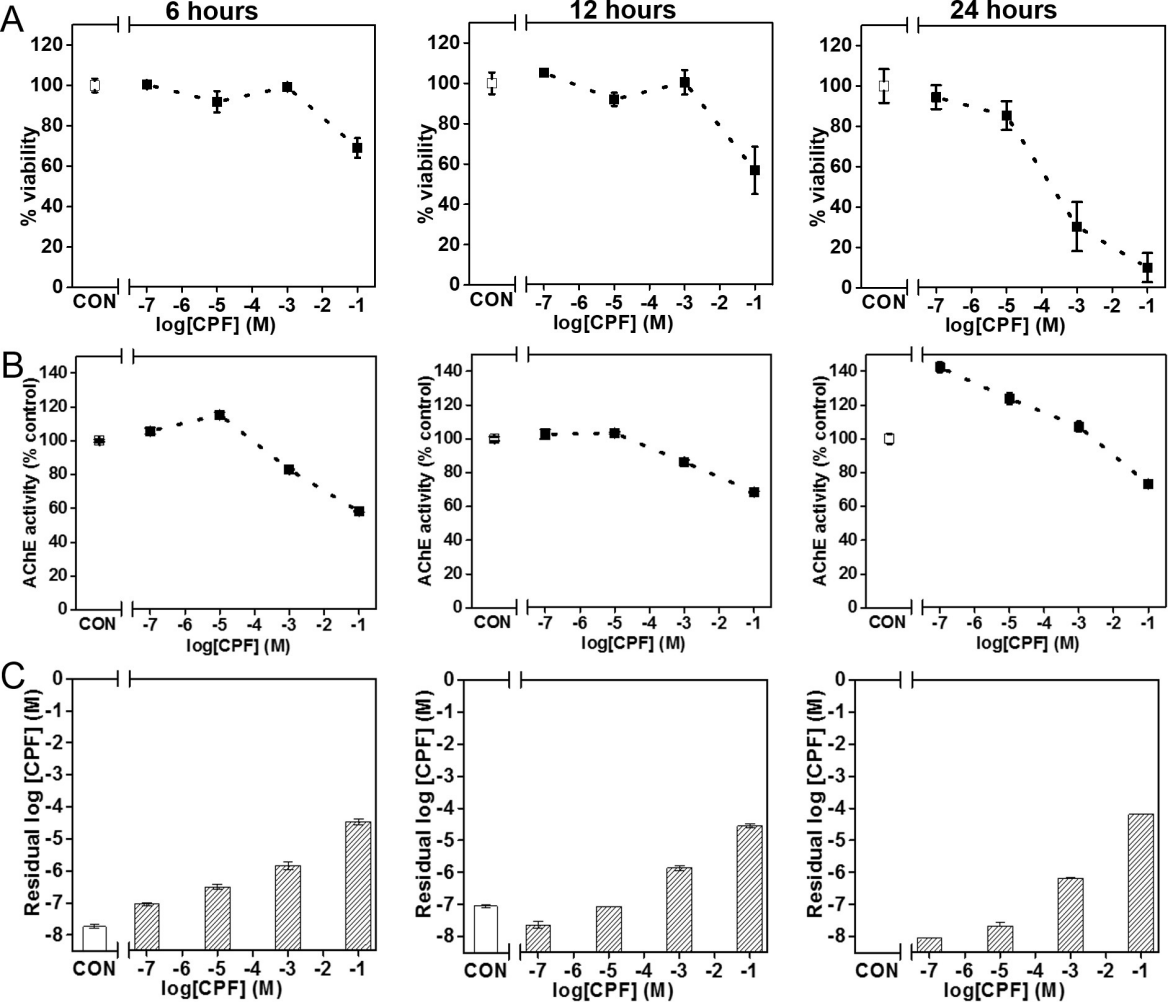
Time-dependent viability(A), AChE activity (B), and residual CPF concentration (C) measured in 3D BMPS platform after varying concentrations of CPF exposure.

**Fig 3:**
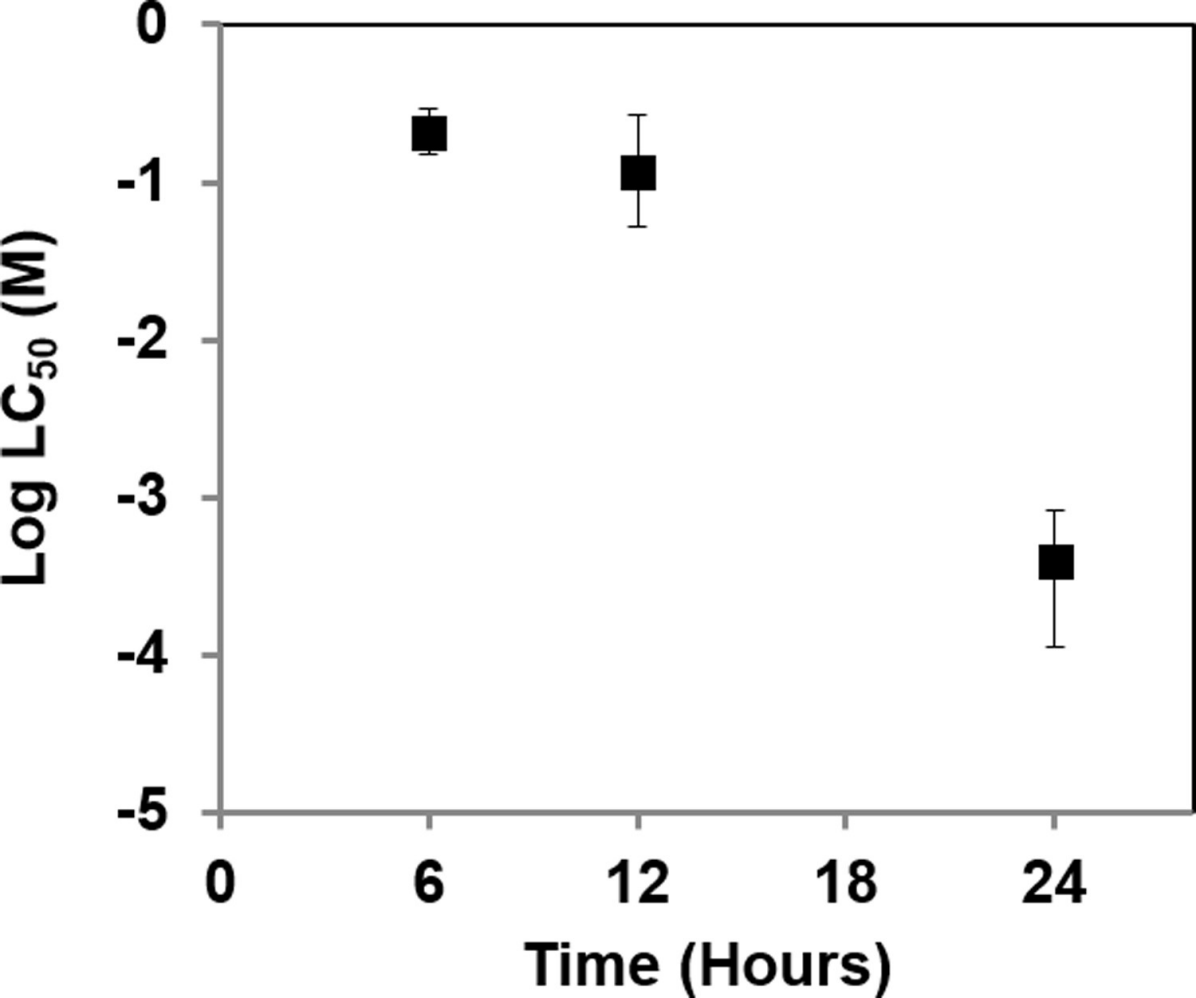
Viability (LC_50_) after CPF exposure at varying time points (hours) using 3D BMPS platform. The error bars were shown as 95% confidence intervals (CI) (profile likelihood).

Using the BMPS platform, we tested the toxicity of CPF at concentrations of 10^−1^, 10^−3^, 10^−5^, and 10^−7^ M for 6-, 12-, and 24-hour exposure times. Cell viability, AChE activity, and residual CPF concentration were measured. As shown in Fig 2, cell viability and AChE activity decreased with an increase in CPF concentration and exposure time (Figs 2A and 1B). Residual CPF also increased with an increase in CPF concentration (Fig 2C). The results show that after 6 and 12 hours of exposure, viability decreased from 100% to 60% with an increase in CPF concentration, and at 24 hours, viability dramatically decreased from 100% to 10% (Fig 1A). AChE activity was also greatly impacted after 24 hours (Fig 2B). Fig 3 shows viability (lethal concentration 50%, LC_50_) decreased with an increase in exposure time.

### OP toxicity with 24-hour exposure times

**Fig 4:**
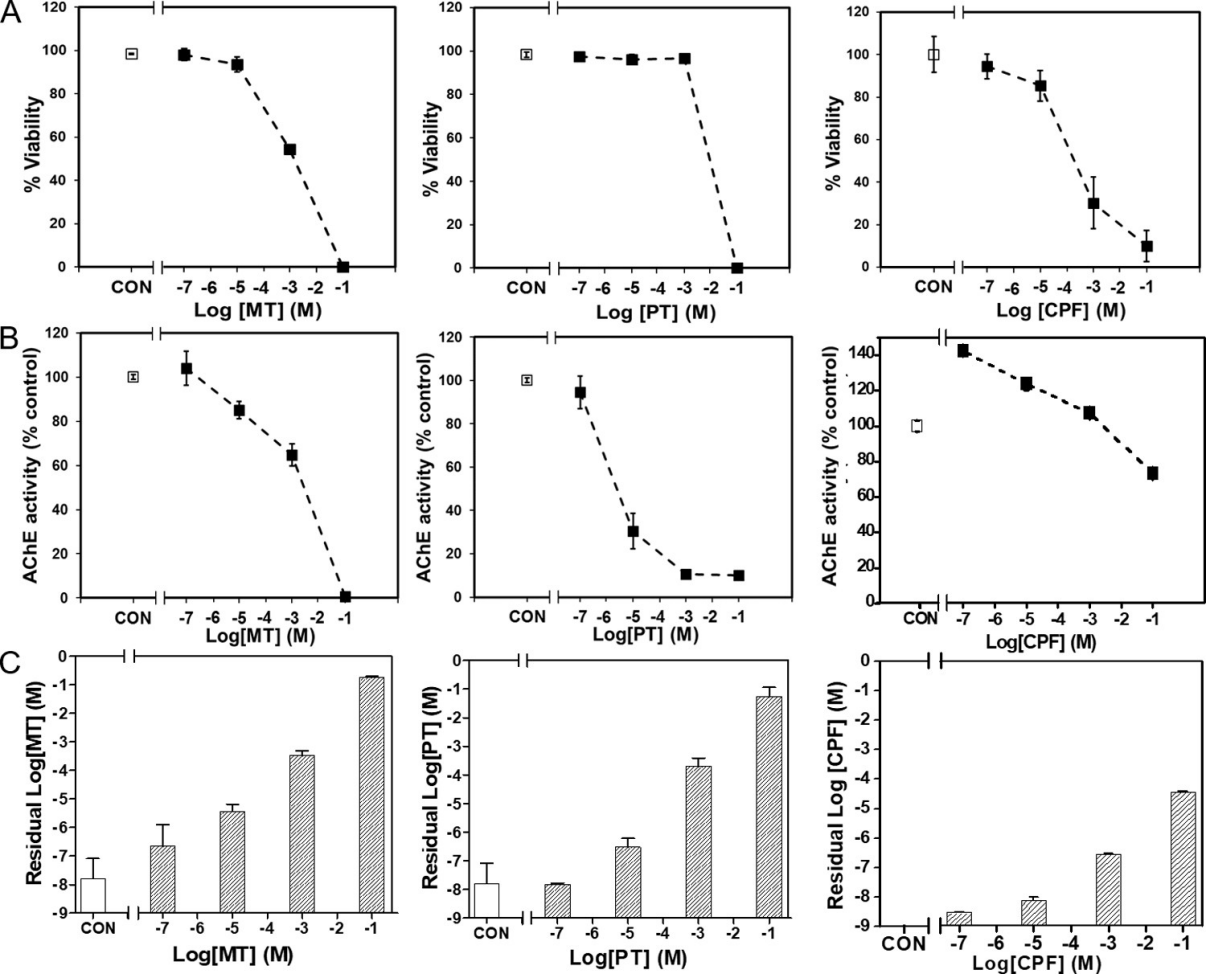
OP (MT, PT, and CPF) exposure results with 3D BMPS model. (A) Viability, (B) AChE activity, and (C) OP residual concentration data. Controls (without OP treatment) are labeled as CON.

We further tested CPF, MT, and PT at four different concentrations (10^−1^, 10^−3^, 10^−5^, and 10^−7^ M) using the BMPS platform. We measured cell viability, AChE activity, and residual concentration after 24 hours of exposure. The results show that CPF, MT, and PT all penetrated through the BBB and inhibited AChE activity, inducing cell toxicity in the brain tissue construct. The cell viability (Fig 4A) and AChE activity (Fig 4B) of the brain lane decreased with an increase in OP concentration for all three OPs. There was a greater decrease in the AChE activity of cells treated with MT and PT compared to cells treated with CPF. The results show that both MT and PT had higher residual concentrations than CPF (Fig 4C).

### *In vitro-in vivo* data correlation

**Fig 5:**
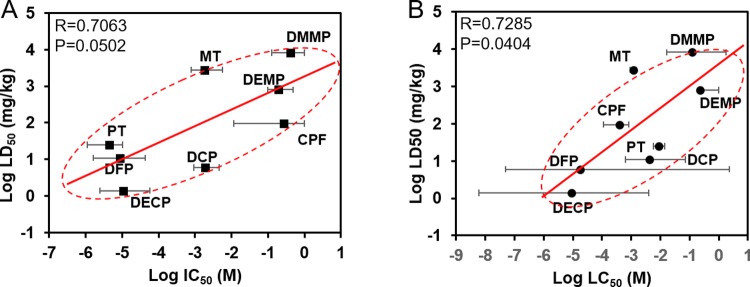
*In vitro* AChE activity and viability correlation with *in vivo* LD_50_ data. A. Estimated IC_50_ for *in vitro* AChE activity vs. *in vivo* LD_50_ for DMMP, DEMP, DECP, DCP, CPF, MT and PT (R = 0.7063, P = 0.0502); B. Log-estimated LC_50_ for *in vitro* viability vs. *in vivo* LD_50_ for DMMP, DEMP, DECP, DCP, CPF, MT and PT (R = 0.7285, P = 0.0404).

Our previous study showed an *in vitro*-*in vivo* correlation with available toxicity data for four OPs [4]. In this study, we expanded the set of OPs for a more accurate and complete correlation that includes the previous data. **Table 1**shows a summary of available *in vivo* LD_50_ data and our estimated LC_50_ (viability) and IC_50_ (AChE activity) data [10–14]. As shown in **Fig 5**, our estimated LC_50_ and IC_50_ show good correlation with *in vivo* data. Based on our *in vitro* BMPS model and *in vivo* data from the literature [15], PT induces greater neurotoxicity than MT and CPF.

**Table 1:** Comparison of *in vitro* 3D BMPS model data with *in vivo* data for OP toxicity.

Chemical	AChE activity (IC_50_, M) (95% CI)	Toxicity (LC_50_, M) (95% CI)	*In vivo* (LD_50_,mg/kg) (toxicity source)
Chlorpyrifos (CPF)	2.735E-1 (7.634E-4 ~ noncomplete CI)	3.949E-4 (1.118E-4 ~ 8.145E-4)	96 (Oral-human)
Malathion (MT)	1.805E-3 (7.670E-4 ~ 5.600E-3)	1.171E-3 (9.216E-4 ~ 1.494E-3)	369 (Oral-rat)
Parathion (PT)	4.338E-6 (1.113E-6 ~ 1.036E-5)	8.924E-3 (5.727E-3 ~ 1.409E-2)	25 (Oral-human)
Diisopropyl fluorphosphate (DFP)	1.863E-3 (9.180E-4 ~ 4.635E-3)	1.781E-5 (5.007E-8 ~ 2.251)	6 (Oral-rat)
Dimethyl methylphosphonate (DMMP)	4.159E-1 (1.253E-1 ~ noncomplete CI)	1.217E-1 (1.651E-2 ~ 1.763)	8210 (Oral-rat)
Diethyl methylphosphonate (DEMP)	1.971E-1 (9.826E-2 ~ 4.933E-1)	2.307E-1 (2.415E-1 ~ noncomplete CI)	800 (Intraperitoneal-mouse)
Diethyl cyanophosphonate (DECP)	1.075E-05 (2.475E-6 ~ 5.813E-5)	8.948E-6 (5.919E-9 ~ 3.906E-3)	1.4 (Intraperitoneal-mouse)
Diethyl chlorophosphate (DCP)	8.688E-06 (1.603E-6 ~4.302E-5)	4.094E-3 (6.202E-4 ~ 7.146E-2)	11 (Oral-rat)

### Toxicity of chemical agents that interact with GABA and/or opioid receptors

**Fig 6:**
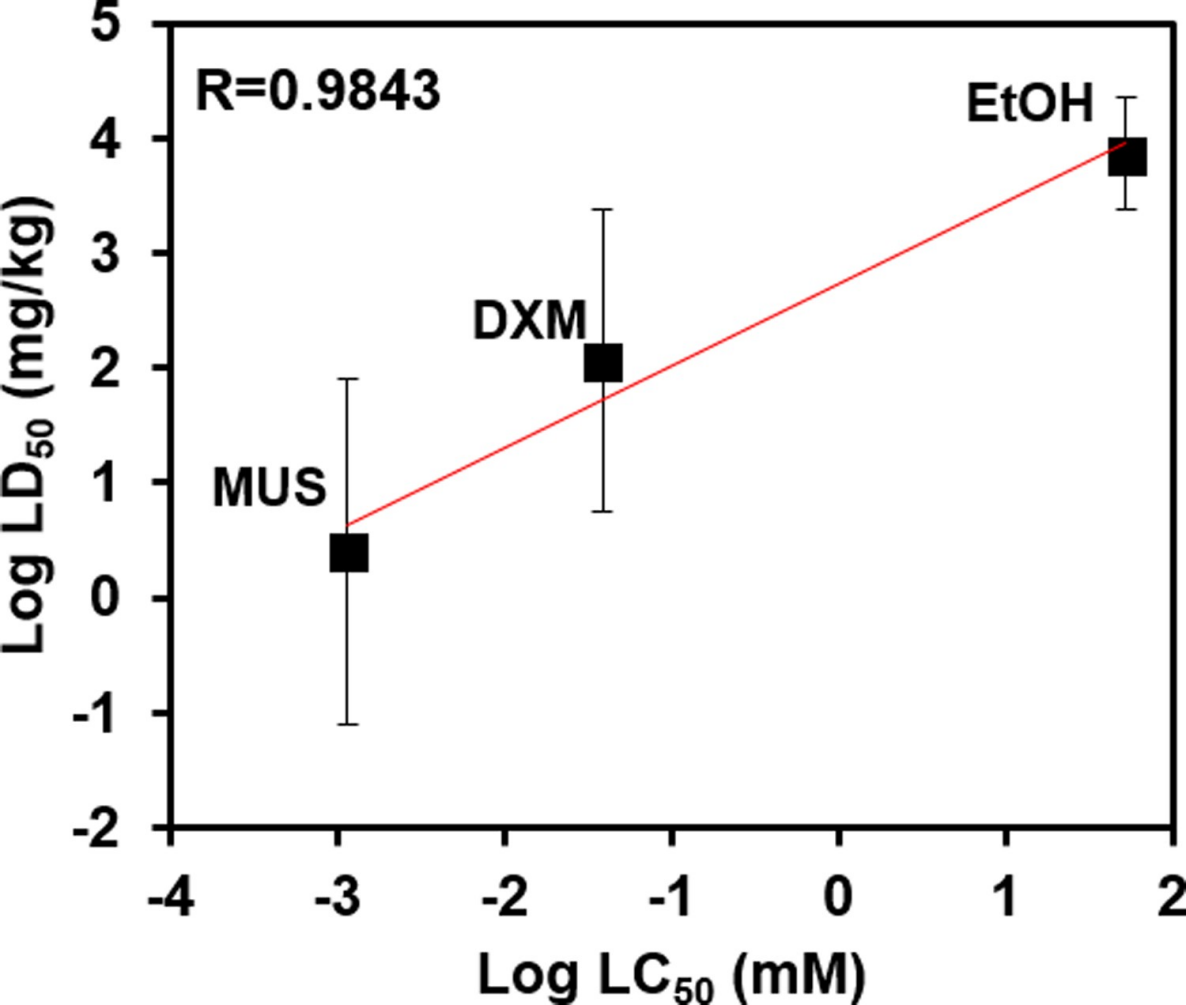
Correlation between *in vitro* viability and *in vivo* LD_50_ data. Log-estimated LC_50_ for *in vitro* viability vs. *in vivo* LD_50_ for MUS, EtOH, and DXM (R = 0.9843).

We screened the toxicity of selected chemical agents MUS and DXM at concentrations of 10^−1^, 10^−2^, 10^−3^, and 10^−4^ mM and EtOH at concentrations of 500, 100, 20, 5 mM, which were added to the blood lane of the brain tissue chips. After 24 hours of exposure, we found that viability decreased with an increase in concentration for all agents (S1 Fig). MUS decreased viability to a greater extent than DXM at the same concentration. Results also showed that EtOH is much less toxic to the brain compared to MUS and DXM, as it induced similar toxicity to MUS and DXM even though it was added at much higher concentrations. Correlation of our viability data (LC_50_ and LD_50_) with available *in vivo* data for MUS [16], DXM [17], and EtOH [18] was summarized in Table 2. As shown in Fig 6, there is a linear correlation between *in vivo* data and our *in vitro* data from the BMPS model (R = 0.9843).

**Table 2:** Comparison of *in vitro* 3D BMPS model data with *in vivo* LD_50_ data on MUS, DXM, and EtOH toxicity.

Chemical	Toxicity (LC_50_, mM) (95% CI)		*In vivo* (LD_50_, mg/kg) (toxicity source)	
MUS	1.159E-3	(3.592E-5 ~3.677E-2)	2.5	(Oral-mouse) [16]
DXM	3.863E-2	(1.860E-3 ~ 8.089E-1)	116	(Oral-rat) [17]
EtOH	5.240E1	(1.797E1 ~ 1.654E2)	7060	(Oral-rat) [18]

### *In silico* PBPK/PD model

**Fig 7:**
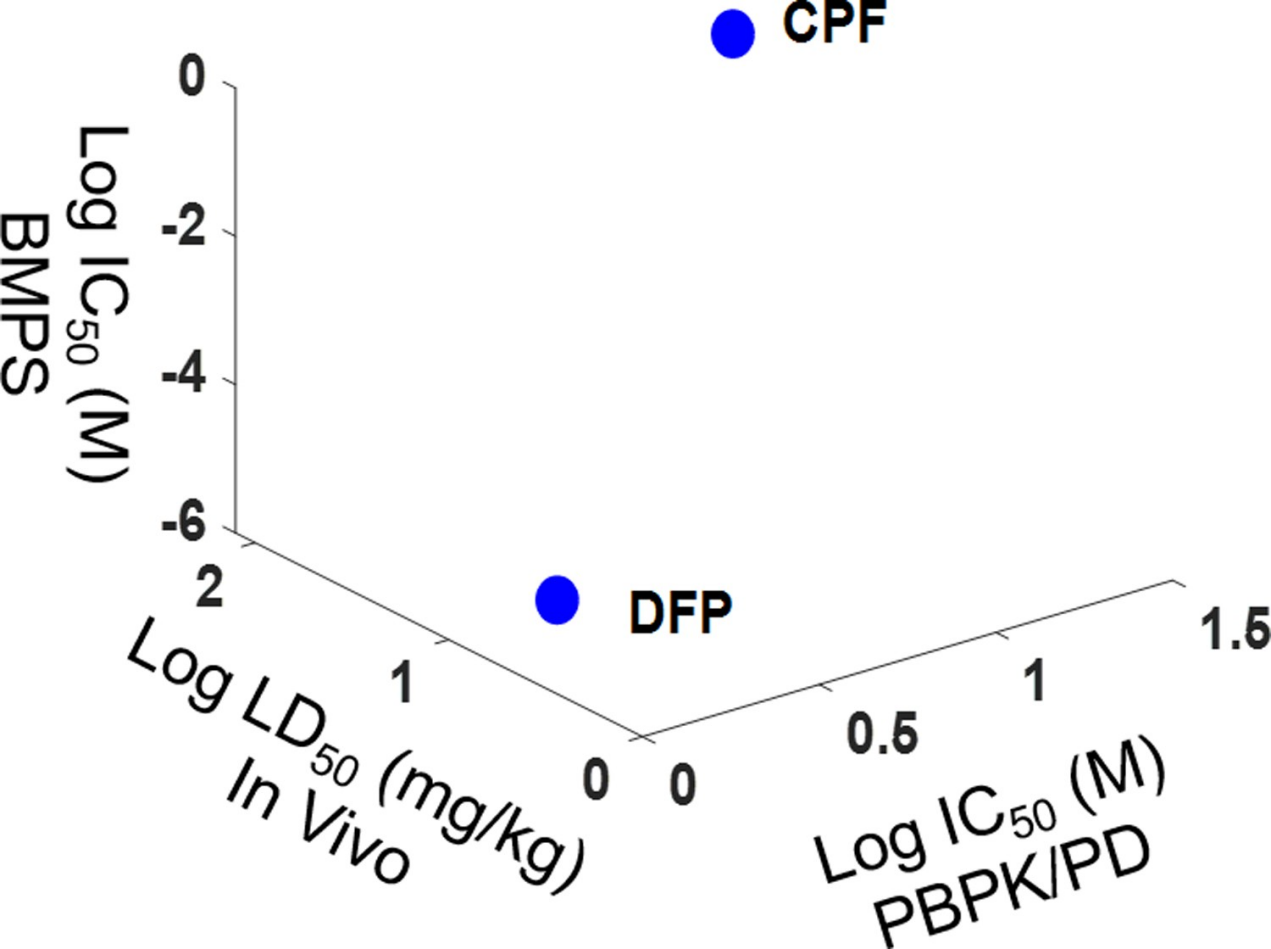
Correlation between *in silico* PBPK/PD model IC_50_, *in vitro* BMPS IC_50_, and *in vivo* LD_50_ data.

A PBPK/PD model was developed for DFP and CPF toxicity based on the Gearhart and Timchalk paper [6, 7]. The PBPK/PD model for DFP and CPF was validated in terms of AChE inhibition and OP concentration change. S2 Fig shows AChE concentration in the blood and brain after IV injection of DFP. AChE increased immediately and decreased in a very short time, ~ 2 minutes, which correlates with existing experimental data in the literature. Next, CPF exposure via IV injection was simulated using a whole-body PBPK/PD model. S3 Fig shows AChE activity after CPF exposure at concentrations of 40 mg/kg, 20 mg/kg, and 1 mg/kg, with results also correlating with previous literature [7]. The results were further correlated with available *in vivo* data [7, 19, 20] and our *in vitro* data from the BMPS platform. Fig 7 shows the correlation between *in vivo* LD_50_ data and our PBPK/PD IC_50_ data, which shows good agreement. All results confirmed that DFP is more toxic than CPF.

## Discussion

Chemical warfare agents and pesticides, including agonists and antagonists of neurotransmitters, can disrupt the CNS and mediate neurotoxicity. In this study, we constructed a BMPS (Fig 1) consisting of 1) ECM-embedded brain cells (neurons, astrocytes, and microglia) and 2) a membrane-free endothelial cell vascular structure with dynamic medium perfusion. This BMPS platform simulated cell-to-cell crosstalk in ECM and BBB physiology in a high-throughput manner [21]. In our previous study, we used this platform to evaluate four OPs for concentration-dependent neurotoxic effects [4]. In this paper, we further validated the BMPS model with an expanded set of OPs and chemical agents that interact with GABA and/or opioid receptors. All the chemicals, including OPs (MT, PT, and CPF) and MUS, DXM, EtOH, penetrated through the simulated BBB and induced neurotoxicity in the ECM-embedded brain cells. For OPs, we tested CPF first to determine its time-dependent toxicity (Figs 2 and 3). As expected, cell viability and AChE activity decreased with an increase in CPF exposure time. Similarly, we performed 24-hour exposures with CPF, MT, and PT and measured OP-induced toxicity. OP toxicity was concentration-dependent and comparable between OPs. The results indicate that CPF is less toxic than MT and PT (Fig 4A and 4B). The residual OP concentration data showed that CPF penetrated the BBB to a greater extent than MT and PT (Fig 4C). In the *in vivo*-*in vitro* correlation analysis, a large population of tested samples further validated the reliability of this platform [22]. *In vitro* results from the BMPS positively correlate with *in vivo* toxicity. The mechanisms of OP toxicity are not limited to AChE activity inhibition, as they also include other mechanisms, such as glutamate-mediated excitotoxicity, diazion-induced apoptotic neuronal death [23], and mitochondrial-mediated and caspase-regulated apoptosis [24–27]. This also explains the extreme descent in viability but mild decrease even some increase in AChE activity when exposed to Ops (e.g. Fig 3, CPF viability and AChE activity). The BMPS platform we explored here can possibly be a useful model to study complex physiology and toxicity mechanisms. Furthermore, this platform can provide knowledge about the differences between *in vitro* and *in vivo* models for neurotoxicology [28].

We tested the toxicity of MUS, DXM, and EtOH at different concentrations, which were determined according to previous studies [29–31]. Cell viability decreased to a greater extent following MUS exposure compared to DXM at the same concentration, indicating that MUS is more toxic than DXM (Fig 6, R = 0.9843). Much higher concentrations of EtOH were needed to attain similar viability results, indicating that EtOH has a much lower toxicity than MUS and DXM. EtOH and MUS are agonists of GABA receptors and the effects of EtOH are not as severe as MUS. The correlation between our viability data and *in vivo* LD_50_ data is with a high coefficient in the linear correlation (R = 0.9843). DXM has inhibitory effects on glutamate-induced neurotoxicity as a NMDA receptor antagonist [32]. Previously, researchers have studied the neuroprotective effect of DXM [32, 33], and our toxicity screening study provides a potential dose–dependent model for the toxic or protective effects of DXM. The common mechanism of MUS, EtOH, and DXM is that they inhibit signal transmission and decrease neuron activity, thus disrupting the CNS [34–36]. In regards to this mechanism, MUS is more potent than DXM and EtOH at similar and lower concentrations.

The correlation between available *in vivo* LD_50_ data and our simulated AChE IC_50_ data for DFP and CPF was used to validate our *in silico* PBPK/PD model. The correlation shows good agreement between *in vivo* data and our *in vitro* BMPS data (Fig 7). A comparison of both compounds shows that DFP is more toxic than CPF, which is consistent with previous literature [4]. As more data for the parameters of the PBPK/PD model become available, other chemical agents can be validated in the future.

We confirmed that the BMPS is a feasible, repeatable, and reproducible platform for toxicity screening of chemical agents. The cell viability and AChE activity results from this platform show positive correlation with available *in vivo* toxicity data. Thus, this platform can be used to reliably predict the *in vivo* effect, toxic or beneficial, of other candidate compounds.

## Supporting information

S1 Fig Viability (%) of brain lane with treatment of MUS (A), EtOH (B) and DXM (C) for 24 hours.
(TIF)

S1 Table Model parameters for AChE inhibition for DFP and CPF. The parameters are cited from literature [19].
(DOCX)

S2 Table Other parameters for PBPK/PD of DFP and CPF modelling in rat brain. The parameters are cited from literature [19].
(DOCX)

S2 Fig Time-course of plasma and brain AChE activity (mg/mL) after IV injection of 1 mg/kg DFP simulated by *in silico* PBPK/PD model. A. Short term within 30 minutes; B. Intermediate term within 1 day; C. Long term over 200 hours.
(TIF)

S3 Fig AChE activity in brain from whole-body PBPK/PD model under CPF, exposure of 40 mg/kg (A), 20 mg/kg (B), and 1 mg/kg (C) within 1 day.
(TIF)
