# Supplementary figures and images for: Three-dimensional (3D) brain microphysiological system for organophosphates and neurochemical agent toxicity screening
Source: PLoS One. 2019 Nov 8;14(11):e0224657. doi: 10.1371/journal.pone.0224657 (PMC6839879; doi:10.1371/journal.pone.0224657)

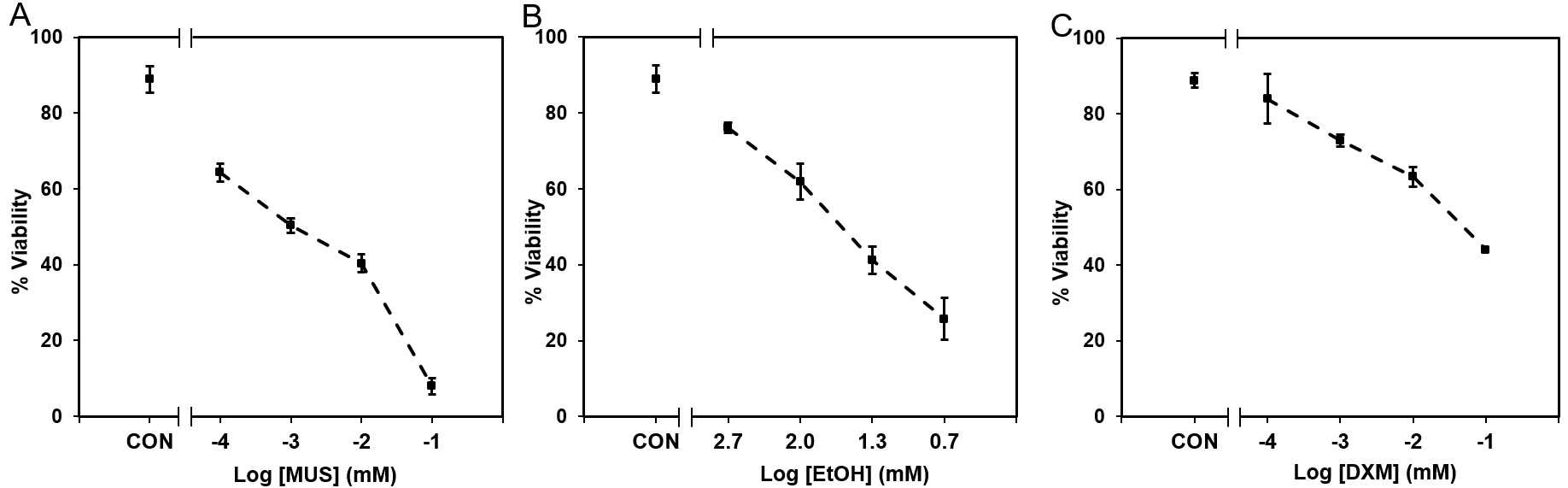

Supplement: S1 Fig — Viability (%) of brain lane with treatment of MUS (A), EtOH (B) and DXM (C) for 24 hours. (TIF) [file pone.0224657.s001.tif]

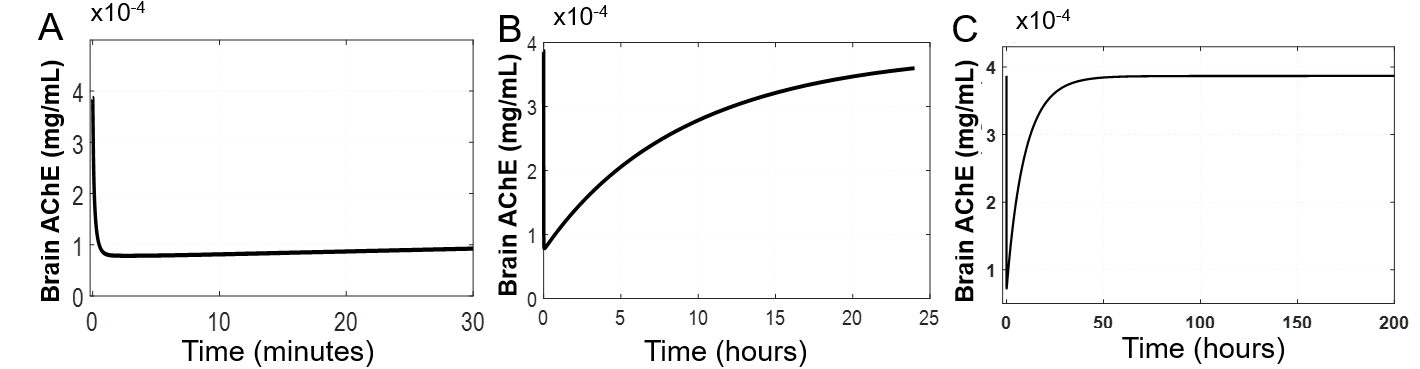

Supplement: S2 Fig — A. Short term within 30 minutes; B. Intermediate term within 1 day; C. Long term over 200 hours. (TIF) [file pone.0224657.s004.tif]

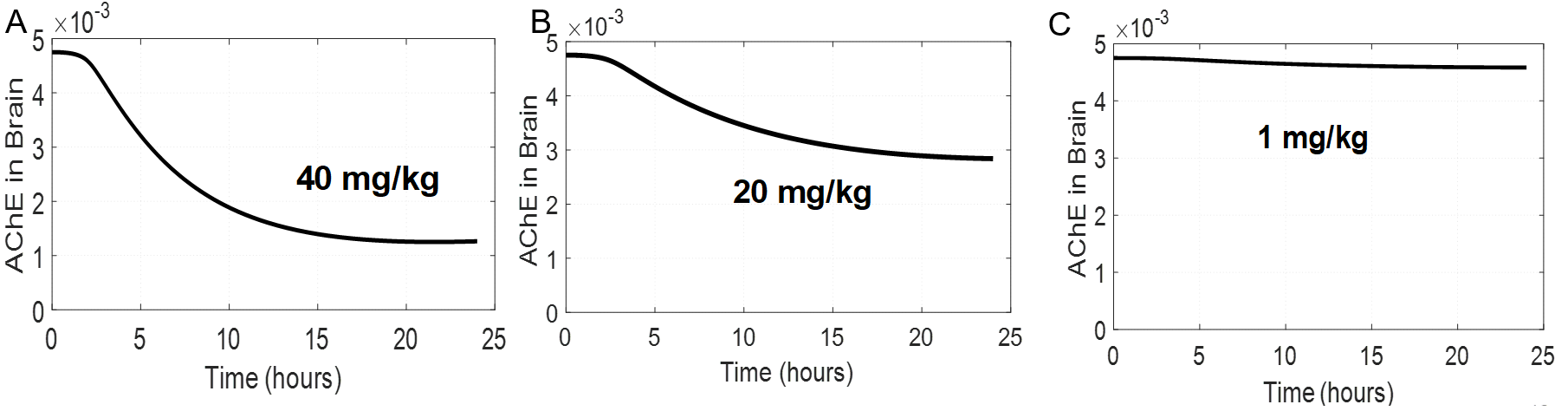

Supplement: S3 Fig — AChE activity in brain from whole-body PBPK/PD model under CPF, exposure of 40 mg/kg (A), 20 mg/kg (B), and 1 mg/kg (C) within 1 day. (TIF) [file pone.0224657.s005.tif]
