# Supplementary material for: Three-dimensional (3D) brain microphysiological system for organophosphates and neurochemical agent toxicity screening
Source: PLoS One. 2019 Nov 8;14(11):e0224657. doi: 10.1371/journal.pone.0224657 (PMC6839879; doi:10.1371/journal.pone.0224657)
Supplement: S1 Table — The parameters are cited from literature [19]. (DOCX) [file pone.0224657.s002.docx]

Supplementary information

Table S1 Model parameters for AChE inhibition for DFP and CPF. The parameters are cited from literature (19).

| Parameters | Rat | | Unit | Description |
| --- | --- | --- | --- | --- |
|  | DPF | CPF |  |  |
| K1 (AChE Inhibition) | 14.16 | 243 | µM-1·hr-1 | All tissue for DPF, other tissues except Red blood cells (RBC) for CPF |
| K2 (AChE Reneneration) | 0.016 | 0.0143 | hr^-1^ | Other tissues except RBC |
| K3 (AChE Aging) | 0.263 | 0.0113 | hr^-1^ |  |
| K4 (AChE Degradation) | 0.010 | 0.010 | hr^-1^ | Brain |
| K5 (AChE synthesis) | 0.0014 | 0.0014 | nmole∙hr^-1^ | Brain |
