# Supplementary material for: Three-dimensional (3D) brain microphysiological system for organophosphates and neurochemical agent toxicity screening
Source: PLoS One. 2019 Nov 8;14(11):e0224657. doi: 10.1371/journal.pone.0224657 (PMC6839879; doi:10.1371/journal.pone.0224657)
Supplement: S2 Table — The parameters are cited from literature [19]. (DOCX) [file pone.0224657.s003.docx]

Supplementary information

Table S2 Other parameters for PBPK/PD of DFP and CPF modelling in rat brain. The parameters are cited from literature (19).

| Rat Brain | Voulme (ml) | Blood flow (ml·hr-1) | Vmax (mg·hr-1) | Km (mg·ml-1) | Partition Cofficient |
| --- | --- | --- | --- | --- | --- |
| DFP | 2 | 137.1 | 9.18 | 0.4398 | 0.67 |
| CPF | 2 | 137.1 | - | - | 0.67 |
